# Supplementary material for: Variability in deer diet and plant vulnerability to browsing among forests with different establishment years of sika deer
Source: PeerJ. 2021 Sep 17;9:e12165. doi: 10.7717/peerj.12165 (PMC8451445; doi:10.7717/peerj.12165)
Supplement: Supplemental Information 1 — Appendix 1–3, Table S1, S3, Figure S1, S2. [file peerj-09-12165-s001.docx]

**Appendix 1: Methods of preparing trnL P6 loop region reference database**

We sampled the leaves of the 59 plant species which were newly sequenced in this study and stored at -30℃ and extracted DNA from the leaves using a modified CTAB (cetyltrimethyl ammonium bromide) method (Milligan 1992). The universal primer pair c-d (Taberlet et al. 1991) was used for the PCR amplification of the whole chloroplast trnL (UAA) introns. The amplification mixture contained 20 ng of the extracted DNA, 12 μl of 2× Multiplex PCR Master Mix, and 0.2 μmol/L of each primer pair. The mixture was denatured at 94°C for 15 min, followed by 35 cycles of 94°C for 1 min, 57°C for 1 min, 72°C for 1 min, and a final cycle of 72 °C for 4 min. Sequencing was performed using the Big Dye Terminator v1.1 Cycle Sequencing Kit (Applied Biosystems, USA) according to the standard protocol. The sequences were obtained from the Akita Prefectural University Biotechnology Center. Plant taxa that shared the same sequences were grouped in lowest taxonomic levels. The taxonomic discrimination rate of the database was calculated by dividing the number of taxa (species, genus, and family) with unique sequences by the total number of taxa included in the database.

The trnL P6 loop database was obtained for 297 plant taxa belonging to 201 genera and 84 families (Table S2). Of the 297 sequences of the P6 loop database, 233 unique sequences were identified, and 35 sequences were newly identified from the newly sequenced 59 samples (Accession no. LC586452-LC586485, LC586838). The sequence fragment lengths ranged from 60-150 bp. The discrimination rate at the species level was 77%, and at the genus level was 95%. All families were identified using the P6 loop.

**Appendix 2: Methods of diet analysis using DNA barcoding**

Each fecal sample was dried for 3 h at 65 °C and then homogenized after freezing with liquid nitrogen. The food plant DNA was extracted from each of the 20-mg fecal samples in dry weight using the DNeasy Plant Mini Kit (QIAGEN) following the manufacturer’s instructions. The DNA extracts were recovered in a total volume of 100 μL. The amplicon library of trnL P6 loop sequence was generated by 2-step PCR as per methods described by a previous study (Hao et al., 2019). The amplification mixture contained 50 ng of the extracted DNA, 0.1 μL of ExTaq, 1μL of ExTaq buffer, 0.8μL of dNTPs, and 400 μmol/L of the universal primer pair g (5′-GGGCAATCCTGAGC CAA-3′) and h (5′-CCATTGAGTCTCTGCACCTATC-3′; Taberlet et al. 2007), a part of the trnL c-d region fused with Illumina sequencing adapters. Thermal cycling conditions were 94°C for 5 min, followed by 35 cycles of 94°C for 30 s, 55°C for 45 s, 72°C for 30 s, and a final cycle of 72 °C for 7 min. The presence of a PCR product of suitable length was confirmed using the MultiNA Microchip Electrophoresis System (Shimadzu). The PCR products were purified using a modified Agencourt AMPure XP (Beckman Coulter). The second PCR to connect the Illumina sequencing adaptors (P5 and P7) was conducted with thermal cycling conditions of 94°C for 5 min, followed by ten cycles at 94°C for 30 s, 55°C for 30 s, 72°C for 30 s, and a final extension at 72°C for 7 min. The indexed PCR products of the 63 samples were pooled into a single library after purification with the Agencourt AMPure XP (Beckman Coulter) to remove the remaining PCR primers. The pooled library was then subjected to an Illumina MiSeq run (run center: Akita Prefectural University Biotechnology Center with the MiSeq Reagent Nano Kit v2 (300 cycles)).

The software Claident v. 0.2.2017.05.22 (https://www.claident.org) (Tanabe & Toju 2013) was used to separate the sequences into each sample and to exclude sequences of low quality (mean quality value < 27). The quality-filtering sequences were divided into operational taxonomic units (OTUs) at the 99% level. We assigned each OTU to the plant taxon in the reference database we prepared by ncbi-blast v.2.7.1+ (<https://ftp.ncbi.nlm.nih.gov/blast/executables/blast+/2.7.1/>). We excluded sequences with low e-values (< 1.0e-25). To avoid the misidentification of plants due to sequencing errors or contaminations, rare plant taxa that had less than 1% sequence reads in each sample were also removed, which was indicated by the study of feeding trials and DNA barcoding of feces with sika deer (Nakahara et al. 2015).

**Appendix 3: Description of the categorization of the groups reflecting vulnerability of plant species**

Group A comprised 16 plants that were categorized in G1 in the vegetation analysis and G2 or G4 in the MDG (mean damage grade of feeding traces) analysis and/or G1 in the DNA analysis. Plants categorized into group A were browsed in the initial establishment years and showed lower coverage in sites with more than 10 years since deer establishment (11 species were absent in the 1978-year sites). These species are suggested to be highly browsed and intolerant to browsing. Group B comprised 20 plants that were categorized in G2 in the MDG analysis and/or G1 in the DNA analysis, but their coverage was unknown because they were not common in all establishment year sites. Plants in group B were also browsed in the initial establishment year and might be intolerant to browsing. Group C consisted of 14 plants categorized in G1 in the vegetation analysis and G3 in the MDG analysis and/or G2 in the DNA analysis. Plants in group C suffered low browsing levels in the initial establishment years (less than 10 years) but high browsing in the latter years, and showed lower coverage in sites with more than 10 years since deer establishment. Group D comprised 33 plants categorized in G3 in the MDG analysis and/or G2 in the DNA analysis, but their coverage was unknown. Plants in group D also suffered browsing after 10 years of deer establishment. Group E comprised two plants categorized in G2 in the vegetation analysis, MDG analysis, and/or DNA analysis. Plants in Group E were browsed throughout the establishment years but their coverage did not decrease. Group F comprised 9 plants categorized in G2 in the vegetation analysis and G3 in MDG analysis and/or G2 or G3 in DNA analysis. Plants in group F were browsed in the latter years but their coverage did not differ among sites, indicating that plants belonging to either group E or F were tolerant to browsing. One unpalatable plant, *Chloranthus serratus*, was not browsed in any sites except at the 1978-year sites and its coverage did not differ among sites. When the results of the MDG analysis and DNA analysis were inconsistent (e.g., G1 in MDG analysis but G1 in DNA analysis), we prioritized the result of the MDG analysis because it can reflect accumulated browsing, while the DNA analyses reflect the deer diet of a few days.

**References**

Milligan B. 1992. Plant DNA isolation. In: Hoelzel AR ed. *Molecular genetic analysis of populations: a practical approach*: IRL Press, Oxford, 59–88.

Hao L, Okano K, Zhang C, Zhang Z, Lei Z, Feng C, Utsumi M, Ihara I, Maseda H, Shimizu K. 2019. Effects of levofloxacin exposure on sequencing batch reactor (SBR) behavior and microbial community changes. *Science of the Total Environment* 672: 227–238.

Nakahara F, Ando H, Ito H, Murakami A, Morimoto N, Yamasaki M, Takayanagi A, Isagi Y. 2015. The applicability of DNA barcoding for dietary analysis of sika deer. *DNA Barcodes* 3: 200-206.

Taberlet P, Gielly L, Pautou G, Bouvet J. 1991. Universal primers for amplification of three non-coding regions of chloroplast DNA. *Plant molecular biology* 17:1105-1109.

Taberlet P, Coissac E, Pompanon F, Gielly L, Miquel C, Valentini A, Vermat T, Corthier G, Brochmann C, Willerslve E. 2007. Power and limitations of the chloroplast trnL (UAA) intron for plant DNA barcoding. *Nucleic Acids Research* 35:e14.

Tanabe AS, Toju H. 2013. Two new computational methods for universal DNA barcoding: a benchmark using barcode sequences of bacteria, archaea, animals, fungi, and land plants. *PLoS ONE* 8: e76910.

Table S1

Geographic locations and attributes of the surveyed sites.

| No. | Site | Altitude (m) | Latitude | Longitude | Deer establishment year | Estimated density (/km^2^) | Surveyed year | Deer feces |
| --- | --- | --- | --- | --- | --- | --- | --- | --- |
| 1 | Suezaki | 122 | 39.04 | 141.69 | 1978 | 7-10 | 2016 | Yes |
| 2 | Akasaka | 363 | 39.16 | 141.72 | 1978 | 15-20 | 2016 | Yes |
| 3 | Kuromoriyama | 675 | 39.20 | 141.69 | 1978 | 15-20 | 2017 | Yes |
| 4 | Ooiwaisawa | 437 | 39.22 | 141.67 | 1978 | 20-25 | 2017 | Yes |
| 5 | Fuefuki | 838 | 39.34 | 141.66 | 2003 | 25-30 | 2016 | Yes |
| 6 | Kiumi | 318 | 39.04 | 141.39 | 2003 | 5-7 | 2016 | Yes |
| 7 | Hirazasa | 525 | 39.26 | 141.50 | 2003 | 10-15 | 2017 | Yes |
| 8 | Kakudesawa | 345 | 39.26 | 141.38 | 2003 | 3-5 | 2016 | No |
| 9 | Kamiayaori | 695 | 39.37 | 141.44 | 2003 | 3-5 | 2017 | Yes |
| 10 | Setamai | 458 | 39.17 | 141.44 | 2011 | 7-10 | 2017 | Yes |
| 11 | Miyamori | 533 | 39.21 | 141.43 | 2011 | 7-10 | 2016 | Yes |
| 12 | Hitokabe | 380 | 39.24 | 141.36 | 2011 | 1-3 | 2017 | Yes |
| 13 | Nakadakiyama | 562 | 39.46 | 141.54 | 2011 | 10-15 | 2016 | Yes |
| 14 | Nagakurayama | 217 | 39.19 | 141.28 | 2014 | 1-3 | 2016 | No |
| 15 | Tamakisawa | 312 | 39.20 | 141.28 | 2014 | 1-3 | 2016 | Yes |
| 16 | Shiwa | 508 | 39.52 | 141.29 | 2014 | 7-10 | 2017 | Yes |
| 17 | Takizawa | 185 | 39.78 | 141.16 | 2014 | 5-7 | 2017 | Yes |
| 18 | Iwami | 203 | 39.74 | 140.41 | 2014 | - | 2016 | No |
| 19 | Minamisawa | 281 | 40.00 | 140.30 | 2014 | - | 2016 | No |
| 20 | Hiyamizusawa | 656 | 40.01 | 140.51 | 2014 | - | 2016 | No |
| 21 | Terasawa | 116 | 40.31 | 140.26 | 2014 | - | 2016 | No |
| 22 | Tazawa | 296 | 39.73 | 140.73 | 2014 | - | 2017 | Yes |

Note. Sika deer density was obtained from the data estimated by the Japanese Ministry of Environment (2017) based on data in 2014. We searched for deer feces across each site (500m × 500m) and the presence of deer feces is indicated by Yes or No.

Table S3

A list of analyses conducted for each survey.

| No. | Survey | Response variable | Explanatory variables | Unit | Analysis |
| --- | --- | --- | --- | --- | --- |
| 1-1 | Vegetation | No. Species, Diversity Index | Deer establishment year | Site | GLMM |
| 1-2 | Vegetation | community composition | Deer establishment year | Site | PERMANOVA |
| 1-3 | Vegetation | sum of vegetation coverage | Deer establishment year | Plot | GLMM |
| 1-4 | Vegetation | sum of vegetation coverage (per life form and plant architecture) | Deer establishment year | Plot | GLMM |
| 1-5 | Vegetation | Coverage of four establishment years | Life form/ Plant architecture | Species | PERMANOVA |
| 1-6 | Vegetation | Coverage of four establishment years | - | Species | K-means |
| 2-1 | Feeding trace | MDG | Plant coverage | Species | GLMM |
| 2-2 | Feeding trace | MDG of four establishment years | Life form/ Plant architecture | Species | PERMANOVA |
| 2-3 | Feeding trace | MDG of four establishment years | - | Species | K-means |
| 3-1 | DNA barcoding of feces | proportion of number of reads of each plant taxa | Deer establishment years | Sample | PERMANOVA |
| 3-2 | DNA barcoding of feces | proportion of number of reads of each plant category (per life form and plant architecture) | Deer establishment years | Sample | GLMM |
| 3-3 | DNA barcoding of feces | proportion of number of reads of plant taxa in four establishment years | Life form/ Plant architecture | Sample | PERMANOVA |
| 3-4 | DNA barcoding of feces | proportion of number of reads of plant taxa in four establishment years | - | Sample | K-means |

Fig. S1 Vegetation coverage (mean ± SE) per plot of categories of (a) plant life history and (b) plant architecture among sika deer establishment years. Different letters indicate significant differences (P < 0.05) among deer establishment years within each category.

Fig. S2 Proportion of number of DNA reads (mean ± SE) per feces of categories of (a) plant life history and (b) plant architecture among sika deer establishment years. Different letters indicate significant differences (P < 0.05) among deer establishment years within each category.
